# Supplementary material for: Simplicial complex entropy for time series analysis
Source: Sci Rep. 2023 Dec 20;13:22696. doi: 10.1038/s41598-023-49958-6 (PMC10733285; doi:10.1038/s41598-023-49958-6)
Supplement: Supplementary file 1 — Supplementary Figures. [file 41598_2023_49958_MOESM1_ESM.pdf]

Supplementary material
“Simplicial complex entropy for time series analysis”

L. Guzmán-Vargas, A. Zabaleta-Ortega, and A. Guzmán-Sáenz
Unidad Profesional Interdisciplinaria en Ingeniería y Tecnologías Avanzadas, Instituto Politécnico Nacional, 07340 Ciudad de México, México
Topological Data Analysis in Genomics, Thomas J. Watson Research Center, Yorktown Heights, NY USA

We examine the impact of the system size, *N*, on the $SCAE_1$ (and Cross-$SCAE_1$) values for the (i) logistic dynamics, (ii) heartbeat interval time series and (iii) coupled logistic maps. The dependence of $SCAE_1$ on the length of the time series is shown in supplementary figure 1, for the three cases mentioned above.

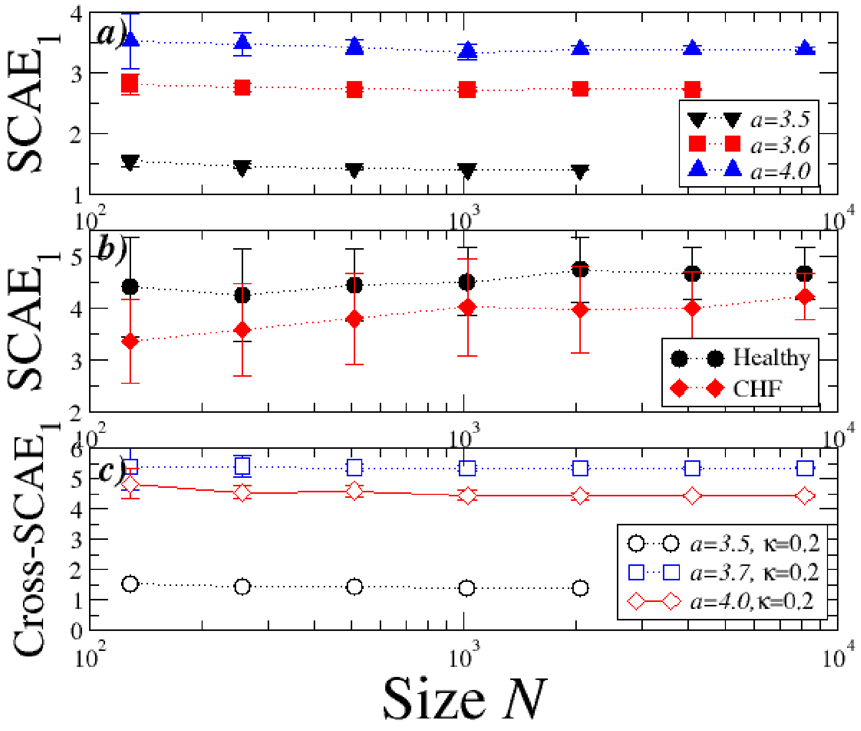

Supplementary figure 1. Dependence of the $SCAE_1$ on the size N. a) SCAE calculations for logistic dynamics and several values of the parameter α. b) SCAE results for heartbeat interval time series from healthy and heart failure gorups. c) Cross-$SCAE_1$ results for coupled logistic maps with coupling parameter κ=0.2 and several value of the parameter α. In all calculations, we used *k*=1, ε=0.1 and d=2. For b) and c), the vertical bars at each value represent the standard deviation of 10 independent realizations, and for b), the vertical bars correspond to the standard deviation from the number of subjects in each group.

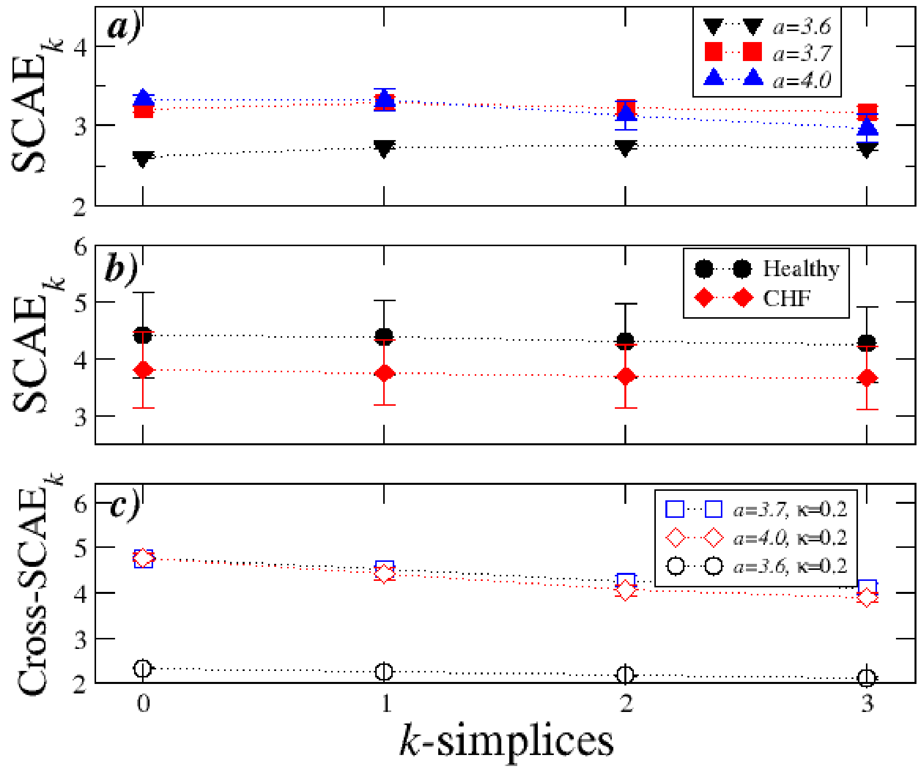

Supplementary figure 2. Dependence of the SCAE on the dimension *k* of the simplices. a) SCAE calculations for logistic dynamics and several values of the parameter α. b) SCAE results for heartbeat interval time series from healthy and heart failure gorups. c) Cross-SCAE results for coupled logistic maps with coupling parameter *κ*=0.2 and several value of the parameter α. In all calculations, we used ε=0.1, *d*=2 and *N*=1024. For b) and c), the vertical bars at each value represent the standard deviation of 10 independent realizations, and for b), the vertical bars correspond to the standard deviation from the number of subjects in each group.

The dependence of $SCAE_k$ on the dimension *k* of the simplices is shown in supplementary figure 2 for the three cases mentioned above.

As shown in supplementary figure 1, for the three cases analyzed, the $SCAE_1$ exhibits slight variations as *N* grows. Also, the $SCAE_k$ shows stable values with respect to the dimension of the simplices (supplementary figure 2).
